# Recent advances in non-alcoholic steatohepatitis-associated hepatocellular carcinoma: immune cells, metabolic dysregulation, and therapeutic strategies

**DOI:** 10.3389/fonc.2025.1744299

**Published:** 2026-01-21

**Authors:** Lisi Liu, Xun Duan, Baozhao Ju

**Affiliations:** 1 School of Basic Medical Sciences, Liaoning University of Traditional Chinese Medicine, Shenyang, Liaoning, China; 2 Shenyang Institute of Automation, Chinese Academy of Sciences, Shenyang, Liaoning, China

**Keywords:** cancer immunotherapy, hepatocellular carcinoma, immunometabolism, non-alcoholic steatohepatitis, regulators, tumor microenvironment

## Abstract

Non-alcoholic steatohepatitis (NASH), the inflammatory progression of non-alcoholic fatty liver disease (NAFLD), is a leading cause of hepatocellular carcinoma (HCC) amid rising obesity and metabolic syndrome. This review elucidates the immunometabolic interplay driving NASH-HCC pathogenesis. Immune cells, including Kupffer cells, monocyte-derived macrophages, and T-cell subsets, orchestrate chronic inflammation and fibrosis via cytokine cascades (TNF-α, IL-1β, TGF-β1) and polarization shifts. Metabolic dysregulation—including insulin resistance, lipid accumulation, and oxidative stress—exacerbates hepatocyte injury, disrupts the balance between apoptosis and compensatory proliferation, and promotes immune evasion through pathways such as β-catenin/TNFRSF19 signaling and hypoxia-inducible factor 1-alpha (HIF-1α). Gut-liver axis alterations further amplify inflammation. Therapeutic advances include immunotherapies (PD-1 inhibitors combined with anti-angiogenics), metabolic regulators (PPARα/FXR agonists, GLP-1RAs), and lifestyle interventions, though NASH-HCC shows reduced immunotherapy efficacy due to unique immunosuppressive microenvironments. Future directions emphasize novel immune targets (MDSCs, SLAMF1), metabolic reprogramming, and microbiota modulation for precision therapies. Integrating multimodal approaches holds promise for halting NASH-to-HCC progression and improving outcomes.

## 1 Introduction

Non-alcoholic steatohepatitis (NASH), the progressive inflammatory form of non-alcoholic fatty liver disease (NAFLD), is a major contributor to chronic liver pathology and a recognized cause of hepatocellular carcinoma (HCC) in developed countries (1–3). NAFLD encompasses a spectrum of hepatic disorders, ranging from benign steatosis to NASH, which is characterized by lobular inflammation, varying degrees of fibrosis, and hepatocellular ballooning (4, 5). The global rise in obesity, type 2 diabetes mellitus (T2DM), and metabolic syndrome, which are significant risk factors for hepatic lipotoxicity and immunological dysfunction, parallels the increasing incidence of NASH-related HCC (6–8).

Epidemiological data show that over 25% of adults worldwide have NAFLD, with NASH responsible for a large share of liver-related illness and death (1, 9). The incidence of NASH and its complications, including cirrhosis and HCC, has risen significantly in regions such as Greater China and Japan. In China, NASH prevalence is estimated at 2.4% to 6.1% (10, 11). The growing number of liver transplants for NASH-induced end-stage liver disease underscores the need for effective diagnostic and treatment strategies (12).

The combination of metabolic load, mitochondrial dysfunction, and immune-mediated inflammation drives the complex pathophysiology of NASH and its development to HCC (2, 13–17). Insulin resistance worsens steatosis and disrupts lipid homeostasis, whereas hepatocyte lipid accumulation causes oxidative stress, endoplasmic reticulum (ER) stress, and pro-inflammatory signaling (14, 15). By interacting with hepatic stellate cells (HSCs) and other non-parenchymal cells, hepatic macrophage activation, including indigenous Kupffer cells and invading monocyte-derived populations, initiates inflammatory cascades and promotes fibrogenesis (18–20). Recent research has also shown the role of other immune cell subsets, including CD8^+^CXCR6^+^ T cells, innate lymphoid cells, and neutrophils, in forming the immunopathological milieu that promotes the development of HCC (21–25). Clarifying the immunometabolic processes underlying NASH-associated hepatocarcinogenesis has become crucial in light of this. Developments in experimental models, non-invasive diagnostics, and therapeutics are converging to improve our understanding of how diseases progress and enable tailored therapies (26, 27). In addition to highlighting existing clinical translation issues, this review summarizes recent findings on the immunological and metabolic interplay in NASH-associated HCC and investigates new treatment approaches aimed at preventing the progression from steatohepatitis to malignancy.

## 2 The role of immune cells in NASH

### 2.1 Composition of intrahepatic immune cells

**Figure 1:**
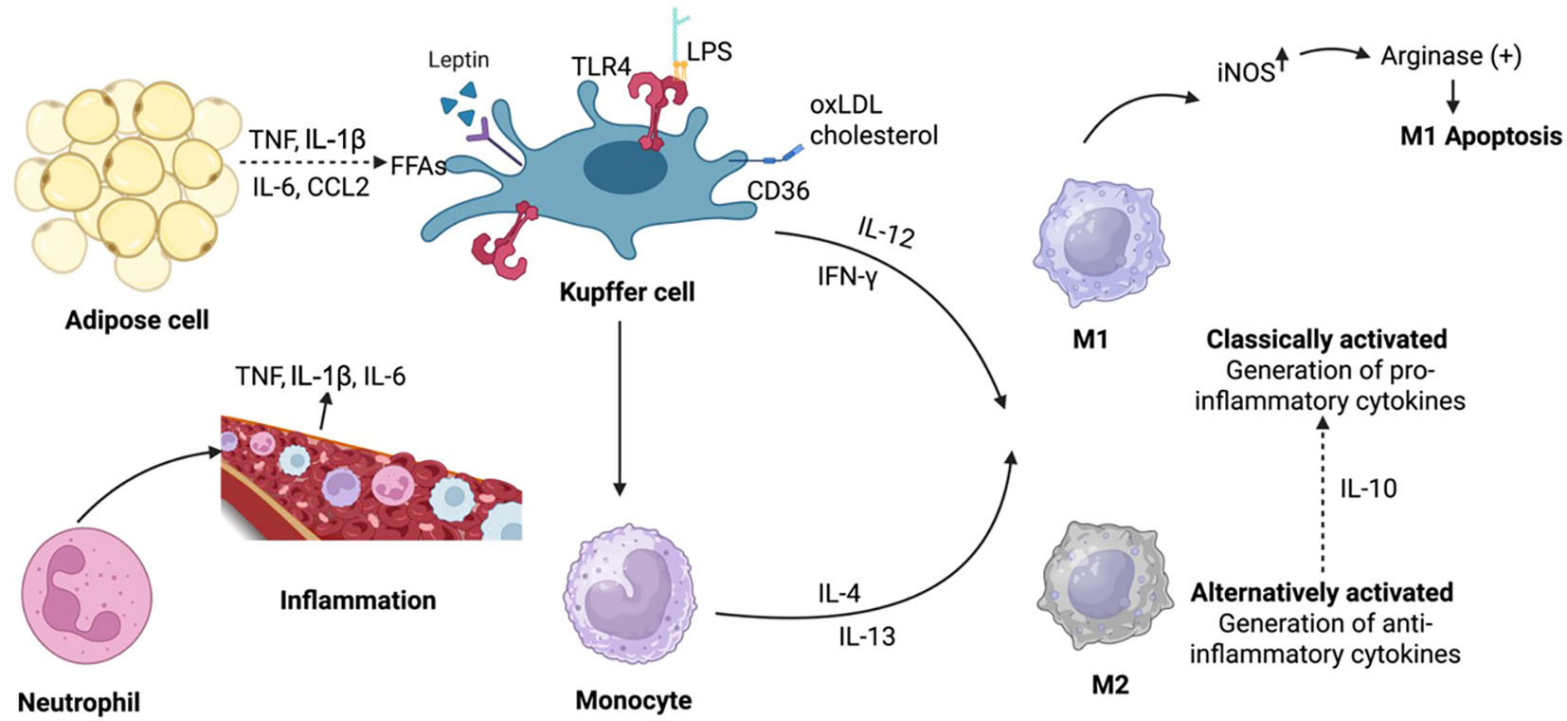
Main NAFLD/NASH macrophage polarization and activation factors. Resident KCs or recruited monocytes produce liver macrophages. Monocytes may be polarized into standard or alternative M1- or M2-type macrophages *in vitro*. In NASH, M1 macrophages cause inflammation whereas M2 macrophages reduce it. M2 macrophages produce IL10, which activates arginase to specifically apoptosis M1 KCs with high iNOS levels. LPS, FFAs, leptin, and cholesterol and oxLDL may activate KCs, FFAs, LEPR from adipose tissue, and CD36 and SRA in NAFLD/NASH. KCs release TNF, IL-1β, and IL-6 to maintain neutrophil balance. In NAFLD, monocytes become M1 macrophages, worsening hepatic inflammation. Key terms: NAFLD, NASH, KCs, IL-10, iNOS, LPS, TLRs, FFAs, LEPR, oxLDL, SRA, TNF, IL-1β.

The intrahepatic immune landscape in NASH is composed of diverse immune populations, including Kupffer cells (KCs), monocyte-derived macrophages (MoMFs), T cells, natural killer (NK) cells, and invariant natural killer T (iNKT) cells, all of which orchestrate disease pathogenesis. Under physiological conditions, KCs maintain immunological tolerance; however, in NASH, they become activated in response to lipotoxic stress and damage-associated molecular patterns (DAMPs), thereby initiating a cascade of pro-inflammatory cytokine release and subsequent immune cell recruitment (28, 29). Concurrently, infiltrating MoMFs differentiate into pro-inflammatory macrophages that engage and activate hepatic stellate cells (HSCs), thereby driving fibrogenic remodeling of the liver. Hepatic damage is exacerbated by a pathological shift in T cell subsets, including dysfunctional regulatory T cells (Tregs) and increased Th1/Th17 cells. CD4^+^ T cell modulation by fatty acids promotes fibrogenesis, while Treg dysfunction drives inflammation and steatosis (30–33). MAIT cells, which are abundant in the liver, mediate both beneficial and harmful reactions when they are dysfunctionally activated in chronic liver disease (34). In NASH-HCC, MAIT cells exhibit considerable metabolic plasticity, with altered glycolytic flux and mitochondrial function influencing their cytokine secretion profiles (35, 36). Upon activation by bacterial-derived riboflavin metabolites via MR1, MAIT cells produce pro-inflammatory cytokines such as IL-17 and IFN-γ, contributing to hepatic inflammation and fibrosis (37, 38). Chronic lipid accumulation and oxidative stress in the NASH liver impair MAIT cell function, shifting them toward a dysfunctional phenotype that paradoxically exacerbates immune dysregulation rather than protecting the tissue (39, 40). Similarly, γδ T cells, which serve as a bridge between innate and adaptive immunity, are increasingly recognized for their role in NASH-driven hepatocarcinogenesis. These cells respond rapidly to non-peptide antigens and produce IL-17A in response to inflammatory cues and free fatty acid accumulation. IL-17–producing γδ T cells promote fibrogenesis by activating hepatic stellate cells and recruiting neutrophils, while also facilitating tumor-promoting inflammation through sustained NF-κB activation (41, 42). Their metabolic programming is tightly linked to fatty acid oxidation (FAO) and mTORC1 signaling, both of which are influenced by the nutrient-rich, lipotoxic hepatic environment (43, 44). Furthermore, group 1 and group 3 innate lymphoid cells (ILCs) infiltrate the NASH liver and are shaped by local metabolic signals, including hypoxia-induced HIF-1α and microbiota-derived short-chain fatty acids (SCFAs) (45, 46). Group 3 ILCs produce IL-22 and IL-17, contributing to epithelial regeneration and inflammation, whereas group 1 ILCs, akin to NK cells, exhibit reduced cytotoxicity under lipid stress. Importantly, IL-15 signaling and altered lipid metabolism regulate ILC survival and function, implicating these cells as metabolically sensitive immunomodulators in the NASH-HCC transition (47–51) (**Figure 1**).

Although both tissue-resident and circulating natural killer (NK) cell subsets actively surveil activated hepatic stellate cells (HSCs) and stressed hepatocytes, their cytotoxic function is attenuated in NASH and negatively correlates with fibrosis severity (52, 53). Invariant natural killer T (iNKT) cells further modulate the inflammatory milieu via cytokine secretion and cell–cell interactions (54). During inflammation, liver sinusoidal endothelial cells (LSECs) act as gatekeepers, promoting immune cell adherence and retention (55). Additionally, the gut-liver axis integrates systemic metabolic signals with local immunity by influencing immunological composition and activation via metabolites produced from the microbiota (56, 57). Microbial-derived metabolites, particularly short-chain fatty acids (SCFAs) and secondary bile acids, can modulate Kupffer cell polarization and T cell differentiation (58–60). SCFAs such as butyrate and propionate, through G-protein coupled receptors (GPR41, GPR43), enhance Treg development and suppress pro-inflammatory Th17 responses, thereby maintaining immune tolerance (61). Conversely, dysbiosis-associated shifts in bile acid composition can impair FXR–TGR5 signaling, promoting Kupffer cell M1 polarization and pro-inflammatory cytokine production (62). Certain pathobionts such as Escherichia coli and Bacteroides species have been linked to increased endotoxemia and LPS translocation, which further activates TLR4–NF-κB pathways in hepatic macrophages, aggravating inflammation and fibrosis (63, 64). Importantly, microbial imbalance has been implicated in resistance to immune checkpoint inhibitors (ICIs) by reshaping the tumor immune microenvironment, such as reducing CD8^+^ T cell infiltration or inducing myeloid-derived suppressor cells (MDSCs) (65–67). Thus, targeting the gut microbiota and its metabolites represents a promising avenue to reprogram intrahepatic immunity and enhance therapeutic responses in NASH-HCC. Single-cell transcriptomic analyses have uncovered profound immune heterogeneity in NAFLD/NASH, identifying distinct T cell and macrophage subsets with context-dependent immunoregulatory or pathogenic roles (68, 69). The interplay between pro-inflammatory and regulatory immune populations orchestrates hepatic inflammation and fibrogenesis, collectively offering a framework for the development of immunomodulatory therapeutic strategies in NASH.

### 2.2 Immune cell activation and inflammatory response

**Figure 2:**
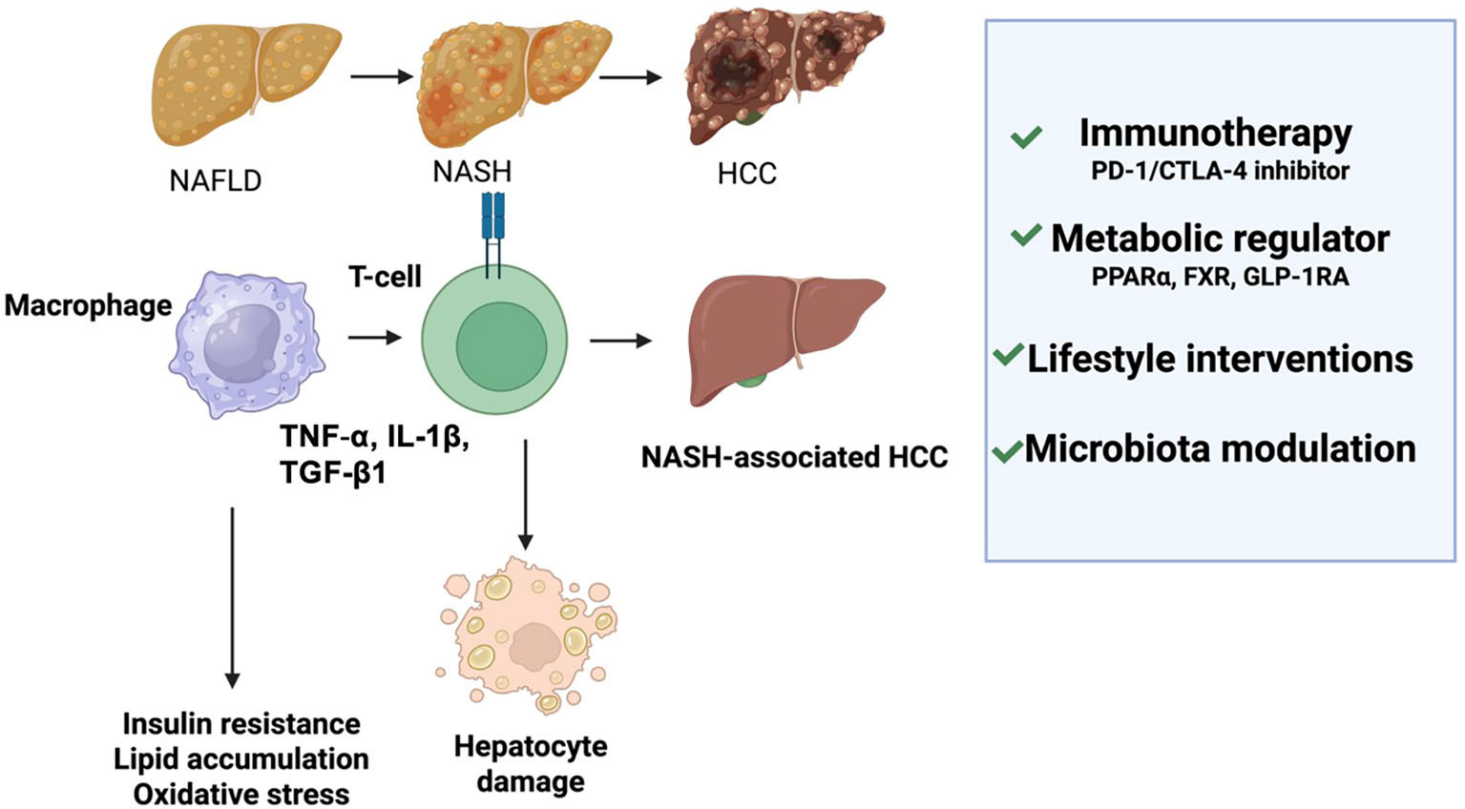
Immune-metabolic dysregulation in NAFLD/NASH-HCC progression and therapeutic strategies. This schematic summarizes how immune and metabolic dysfunction drive immune escape and tumor promotion in NASH-related HCC. On the left, metabolic stress in the NAFLD/NASH microenvironment causes the death of cytotoxic CD4^+^ T cells and expansion of auto-aggressive CXCR6^+^ CD8^+^ T cells, fostering hepatocarcinogenesis. On the right, β-catenin (CTNNB1) mutations activate the TNFRSF19 pathway, suppressing pro-inflammatory cytokines (IL-6, CXCL8) and impairing immune cell recruitment, thereby inducing immune exclusion. Together, these processes coupled with chronic metabolic dysregulation—create an immunosuppressive microenvironment that promotes HCC progression.

Chronic hepatic inflammation is a principal driver of disease progression from NAFLD to NASH, fibrosis, and ultimately HCC. Hepatocyte injury, metabolic stress, and lipotoxicity collectively activate immune cells within the hepatic microenvironment, initiating a cytokine-driven inflammatory cascade (70). Resident Kupffer cells and monocyte-derived macrophages (MoDMacs) recognize gut-derived pathogen-associated molecular patterns (PAMPs) and damage-associated molecular patterns (DAMPs) via pattern recognition receptors (PRRs), including Toll-like receptors (TLRs) and NOD-like receptors (NLRs), leading to activation of NF-κB and inflammasome signaling pathways. This results in the secretion of key pro-inflammatory and profibrotic cytokines such as TNF-α, IL-1β, and TGF-β1 (18, 71–73). Macrophage polarization plays a key role in disease development: M1-like macrophages worsen tissue damage, while M2-like macrophages promote fibrogenesis via TGF-β1 despite their role in tissue healing. This phenotypic equilibrium in KCs is controlled by galectin-12 (71). By producing cytokines that attract immune cells and stimulate hepatic stellate cells (HSCs), neutrophils, NK cells, and ILCs, these cells increase inflammation (74, 75). Adaptive immune responses further aggravate pathology: CD4^+^ and CD8^+^ T cells, including dysfunctional regulatory T cells and T-bet–driven pro-inflammatory subsets, potentiate hepatocellular injury and IFN-γ production (76, 77). Complement-derived C3a and C5a encourage immunological recruitment, but ROS and DAMPs from lipotoxic hepatocytes activate NLRP3 inflammasomes (78–80). By triggering hepatic PRRs and inducing cytokine production, gut-derived endotoxins, such as LPS, worsen liver inflammation (81, 82). The ensuing cytokine milieu (TNF-α, IL-1β, IL-6, and TGF-β1) reinforces a self-reinforcing cycle of inflammation and fibrosis by sustaining immunological activation and initiating HSC-mediated fibrogenesis (18, 83). Notably, the NLRP3 inflammasome serves as a central molecular bridge linking metabolic stress to immune activation. Lipotoxic hepatocytes release ROS and DAMPs, which activate NLRP3 through mitochondrial dysfunction, potassium efflux, and endoplasmic reticulum (ER) stress (84, 85). Upon activation, NLRP3 promotes the cleavage of pro-caspase-1 into active caspase-1, leading to the maturation of IL-1β and IL-18, key amplifiers of hepatic inflammation (86–88). This cascade augments macrophage and neutrophil recruitment and perpetuates hepatocyte damage, fibrosis, and immune cell dysregulation. The intersection of ROS accumulation, lipid peroxidation, and TLR signaling further potentiates NLRP3 activity, situating it at the nexus of metabolic and immunologic injury in NASH (89, 90). Therapeutic targeting of NLRP3—via GPR81 agonists, AMPK activators, or miRNA regulators (miR-137-3p)—has shown promise in attenuating this harmful axis in preclinical studies (88, 91). Targeting immune signaling pathways has become a central focus in emerging therapeutic strategies. Inhibitors of chemokine receptors block immune cell infiltration, while peroxisome proliferator-activated receptor (PPAR) agonists modulate macrophage polarization to shift immune balance. Additionally, phytochemicals such as paeoniflorin and magnoflorine have demonstrated efficacy in dampening inflammatory cascades (92–95) (**Figure 2**).

### 2.3 Polarization and metabolic regulation of immune cells

The polarization of immune cells, especially macrophages, significantly impacts metabolic balance in disease states such as NAFLD and HCC. Glycolysis and oxidative phosphorylation (OXPHOS) are the hallmarks of the pro-inflammatory M1 and anti-inflammatory M2 states that macrophages may adopt due to their metabolic flexibility. While M2 macrophages encourage tissue remodeling via fatty acid oxidation (FAO) and immunosuppression, M1 macrophages increase metabolic inflammation by glycolytic reprogramming and cytokine production (96, 97). In addition to serving as a metabolic substrate, tumor-derived lactate also functions as a signaling molecule that promotes immune evasion by polarizing tumor-associated macrophages (TAMs) toward an M2-like phenotype (98, 99). To fine-tune macrophage fate, molecular regulators such as Sirtuins and lncRNAs integrate metabolic signals with epigenetic regulation (100, 101). Tryptophan metabolism and serine metabolism via IGF1-p38 signaling are two metabolic pathways that directly alter M1/M2 balance, influence inflammatory tone, and influence treatment responses (102, 103). The polarization axis affects fibrotic and inflammatory cascades in hepatic diseases: M1 macrophages worsen damage, while M2 macrophages promote fibrogenesis by releasing TGF-β1 and other mediators. Hepatocyte PPAR-γ–ROS signaling and galectin-12 both influence Kupffer cell fate (71, 104). In addition to macrophages, metabolic reprogramming occurs in neutrophils and dendritic cells, suggesting broader immunometabolic vulnerabilities that may be exploited in cancer and chronic inflammation (105).

## 3 The impact of metabolic disorders on NASH

### 3.1 Insulin resistance

One of the main pathogenic mechanisms in NAFLD and its progressive form, NASH, is insulin resistance (IR). IR disturbs glucose and lipid homeostasis and is characterized by compromised insulin signaling in the liver, adipose tissue, and skeletal muscle. By boosting adipose lipolysis, increasing free fatty acid influx, and promoting *de novo* lipogenesis, IR increases hepatic steatosis in NAFLD, while ironically failing to reduce gluconeogenesis (106, 107). Selective insulin resistance in the liver leads to compensatory hyperinsulinemia and metabolic dysregulation, further disrupting systemic energy balance. The early pathogenic significance of NAFLD is shown by pediatric findings that link its increased incidence with IR and metabolic syndrome (107). Mechanistically, inflammation, oxidative stress, and mitochondrial dysfunction exacerbate IR, which is caused by poor PI3K-Akt signaling and faulty insulin receptor substrate (IRS-1/2) phosphorylation (72, 108). Through mTORC1-SREBP1c activation and compromised insulin signaling, disruption of the hedgehog pathway, especially hepatocyte Smo deletion, causes steatosis and systemic IR (109). Through immunological activation and the involvement of the inflammasome, IR also promotes hepatic inflammation. Notably, activation of the NLRP3 inflammasome increases liver damage by generating IL-1β and IL-18 and disrupts insulin signaling by causing serine phosphorylation of IRS-1. These effects are lessened by GPR81 agonism, making it a potentially useful therapeutic target (110). Even in those who are not obese, IR clinically correlates with NAFLD severity, as indicated by HOMA-IR, TyG, and LP-IR, and shows phenotypic variability (111–113). Genetically driven NAFLD and IR do not seem to be causally related, according to Mendelian randomization experiments, suggesting that the processes are either shared or subtype-specific (114). Through oxidative and lipogenic mechanisms, dietary variables, especially excessive fructose and artificial sweeteners, exacerbate IR and steatosis (115, 116). Insulin-sensitizing drugs (GLP-1R, PPAR, and FXR agonists), lifestyle changes, and new regulators like AMPK, FGL1, and CaSR are examples of therapeutic approaches (117–121). IR is a key target in the treatment of NAFLD and NASH because it coordinates hepatic lipid excess, inflammation, and systemic metabolic imbalance.

### 3.2 Lipid metabolism abnormalities

Lipid metabolic abnormalities play a key role in the development of NAFLD, which leads to NASH, fibrosis, and HCC. Excessive triglyceride and cholesterol buildup is a sign of dysregulated hepatic lipid homeostasis, which impairs hepatocyte function and causes lipotoxicity. Environmental pollutants such as pyrethroids and exposure to high-fat diets (HFDs) have been shown to block β-oxidation regulators (CPT-1) and upregulate lipogenic genes (SREBPs, FAS, and ACC), thereby increasing lipid accumulation and hepatocellular damage (122, 123). ACLY, a crucial link between glycolysis and lipogenesis, drives NASH-HCC progression. In a high-fat/fructose western diet and diethyl nitrosamine injection (WD-DEN) model, ACLY knockout decreased lesion burden, tumor lipid content, and SREBF1 expression by approximately 70%. It also reversed immunosuppression by restoring CD8^+^ T cell IFN-γ secretion and reducing lactate (124). AMPK, a metabolic sensor that, when activated, inhibits lipogenesis and encourages fatty acid oxidation, is essential to these processes (123, 125). Through the LKB1/AMPK axis, substances such as coniferaldehyde help improve lipid and glucose dysregulation (125). AMPK interacts with the NAD^+^-dependent deacetylase SIRT1, forming the AMPK–SIRT1 axis, which promotes mitochondrial biogenesis, autophagy, and anti-inflammatory responses by deacetylating transcriptional regulators such as PGC-1α and NF-κB (126, 127). This signaling network not only facilitates metabolic homeostasis but also alleviates immune exhaustion by enhancing regulatory T cell survival and repressing macrophage M1 polarization (128). Meanwhile, activation of PPARα—a nuclear receptor transcription factor—is tightly coupled to fatty acid oxidation (FAO) and modulates the immune landscape by promoting M2 macrophage polarization, reducing proinflammatory cytokine secretion (TNF-α, IL-6), and restoring immune tolerance (129, 130). Dysfunction of these immunometabolic circuits fosters chronic inflammation and immune evasion in NASH-HCC (131, 132). Together, the AMPK–SIRT1 and PPARα–FAO axes serve as central hubs connecting metabolic stress with immune dysregulation in steatohepatitic carcinogenesis, providing promising therapeutic targets.

Conversely, aberrant activation of liver X receptors (LXR) and suppression of peroxisome proliferator-activated receptor α (PPARα) exacerbate hepatic steatosis and inflammation by disrupting lipid trafficking and immune crosstalk (123, 133). Mitochondrial dysfunction further amplifies lipid imbalance by impairing β-oxidation and promoting reactive oxygen species accumulation and apoptosis (134). In parallel, lysosomal impairment compromises lipid catabolism, thereby aggravating the progression of NAFLD (135). F OXK1, a downstream effector of mTORC1, has also been implicated in promoting steatosis through suppression of fatty acid oxidation (136). Lipid overload mechanistically bridges metabolic stress to immune dysregulation by activating complement cascades, Toll-like receptor (TLR) signaling, and driving macrophage polarization (80, 137). TREM2^+^ macrophages signify an immunometabolic adaptation that attenuates oxidative injury in NAFLD (138). Hepatocellular lipid management and inflammation are regulated by extracellular vesicles (139), and metabolic and inflammatory pathways are further adjusted by genetic variations (APOH) and epigenetic modulators (miRNAs) (140–143). Emerging treatments with translational potential include those that target AMPK, PPARα, or SREBPs (144), as well as natural substances such as resveratrol, sesamin, anthocyanins, and fucoxanthin (145–148). Additionally, interventions targeting autophagy, endoplasmic reticulum stress, and mitochondrial integrity show promise in preclinical models (135, 149).

### 3.3 Inflammation and oxidative stress

Metabolic dysregulation in non-alcoholic fatty liver disease (NAFLD) initiates a self-perpetuating cycle of oxidative stress and inflammation, accelerating fibrosis and hepatocellular damage. Lipid overload—particularly from free fatty acids (FFAs)—exceeds the liver’s antioxidant defenses, leading to lipotoxicity and mitochondrial dysfunction (150, 151). Impaired electron transport chain (ETC) activity further intensifies lipid peroxidation and oxidative injury while triggering pro-inflammatory signaling cascades, notably NF-κB activation. This promotes the upregulation of TNF-α, IL-6, and IL-1β, thereby recruiting hepatic immune populations such as Kupffer cells and infiltrating macrophages (152–154). Inflammation and ROS accumulation are reinforced by insulin resistance associated with metabolic syndrome, which also promotes *de novo* lipogenesis and suppresses fatty acid oxidation (155). Although blockade of the endocannabinoid system exhibits anti-inflammatory potential, CB1 receptor activation paradoxically exacerbates oxidative and nitrosative stress (153).

Multiple regulatory pathways modulate this pathogenic feedback. For example, interventions like Gegen Qinlian Decoction and fucoxanthin attenuate oxidative damage through activation of the AMPK/Nrf2 axis, a key modulator of cellular antioxidant responses (156, 157). MicroRNAs such as miR-137-3p and miR-665-3p further regulate oxidative and inflammatory cascades at the post-transcriptional level (158, 159). In NAFLD, endothelial dysfunction is made worse by chronic intermittent hypoxia, which is frequent in obstructive sleep apnea and exacerbates oxidative damage (160). The transition from basic steatosis to NASH and HCC is facilitated by this reciprocal amplification between ROS and inflammation (161, 162). Therapeutic strategies targeting this axis—such as omarigliptin, probiotics, nobiletin, and resveratrol—have demonstrated efficacy in mitigating hepatic oxidative and inflammatory damage (163, 164).

## 4 The mechanisms linking NASH and hepatocellular carcinoma

### 4.1 Apoptosis and hepatocyte proliferation

Disruption of the delicate balance between hepatocyte death and compensatory proliferation constitutes a critical driver of hepatocarcinogenesis. In diet-induced obesity (DIO) mouse models, a high-fat diet (HFD) induces hepatic steatosis accompanied by elevated oxidative stress, dysregulated insulin and glucose signaling, and aberrant lipid metabolism. These metabolic insults synergistically promote hepatocyte apoptosis, as evidenced by increased caspase-3 activity and upregulation of apoptosis-related genes. Concurrently, hepatocyte proliferation is diminished, indicating a breakdown in the homeostatic coupling of cell death and regeneration (165). This imbalance not only accelerates cellular turnover but also heightens the risk of replication-induced mutagenesis, facilitating the accumulation of somatic mutations. Sustained oxidative stress—central to NASH pathology—further compounds genomic instability through ROS-mediated DNA lesions such as 8-oxoguanine (166, 167). Inflammatory conditions impair DNA repair mechanisms, intensifying mutation load and fostering a pro-tumorigenic environment through the uncoupling of apoptosis and regenerative proliferation (168).

Studies employing genetically engineered mice lacking the pro-survival BCL-2 family protein myeloid cell leukemia 1 have yielded mechanistic insights into hepatocarcinogenesis in NASH. In hepatocyte-specific Mcl1-deficient mice subjected to a NASH-inducing diet, marked increases in hepatocyte death, inflammation, fibrosis, and compensatory proliferation were observed. Notably, these mice developed liver tumors with histological hallmarks of hepatocellular carcinoma (HCC) at significantly higher frequencies than controls, underscoring the paradox whereby excessive hepatocyte apoptosis may drive carcinogenesis in NASH (169). In NASH, the hepatic integrated stress response (ISR) controls hepatocyte fate at the molecular level by inhibiting the somatotroph axis via ATF3, which lowers inflammation and apoptosis while simultaneously reducing hepatocyte proliferation (170). Despite reducing cell death, this suppression worsens fibrosis, underscoring the intricate relationship between apoptosis and proliferation during the course of illness. Moreover, ISR dysregulation under metabolic stress has been implicated in impaired DNA repair and the clonal expansion of mutated hepatocytes, providing an additional pathway to mutagenesis and HCC development (171). Intriguingly, replenishment of NAD^+^ has been shown to restore proliferative capacity and ameliorate liver injury, potentially counteracting ISR-mediated hepatocarcinogenic processes (170).

Several signaling pathways and variables influence this equilibrium. For example, substances such as Ginsenoside Rb1 may reduce HFD-induced hepatocyte apoptosis by activating peroxisome proliferator-activated receptor gamma (PPAR-γ), indicating therapeutic potential for modifying apoptotic pathways (172). On the other hand, environmental pollutants that block the AMPK and PPAR-γ pathways worsen hepatocyte death and steatohepatitis, suggesting that these pathways have protective functions (173). The importance of proliferation in liver repair and carcinogenesis is further highlighted by the fact that proliferating cell nuclear antigen (PCNA), a marker of DNA synthesis and cell proliferation, is downregulated in steatotic livers but can be targeted by agents such as avachinin to promote hepatocyte regeneration and reduce apoptosis (174). Oxidative stress, a hallmark of non-alcoholic steatohepatitis (NASH), also impairs proliferative responses and promotes apoptosis. NADPH oxidases (NOX enzymes) serve as key effectors linking metabolic dysfunction to cell injury by generating reactive oxygen species (ROS) that drive hepatocyte damage (175). Therapeutic antioxidant strategies, including molecular hydrogen administration, have been shown to mitigate steatosis and fibrosis by dampening inflammation and apoptotic signaling (176).

### 4.2 Immune evasion mechanisms

**Figure 3:**
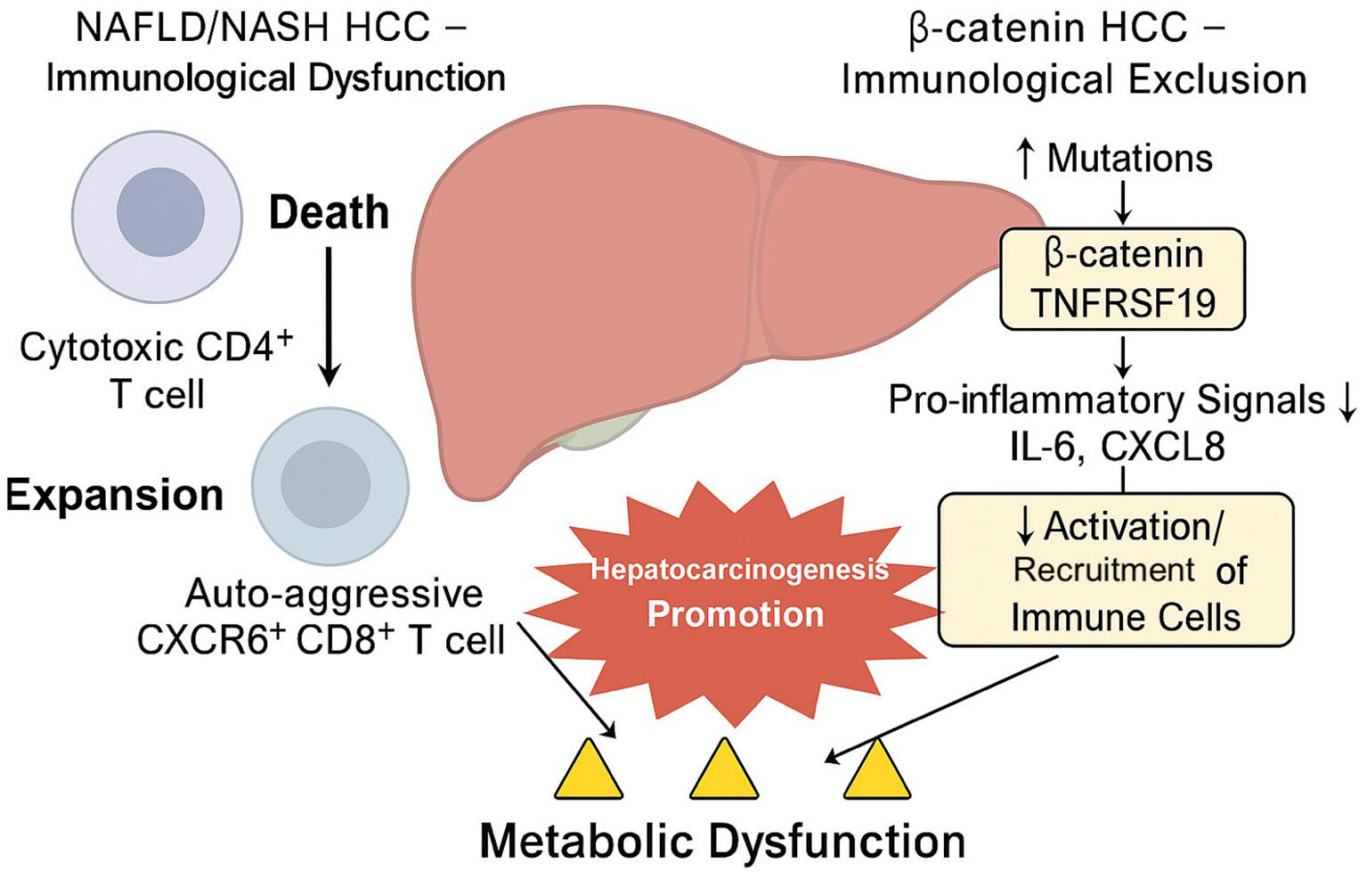
Mechanisms of immune evasion in NAFLD/NASH-associated hepatocellular carcinoma. Liver tumors in NAFLD/NASH evade immunity through β-catenin (CTNNB1)–TNFRSF19–mediated cytokine suppression (IL-6, CXCL8), metabolic stress–induced CD4^+^ T-cell loss, expansion of CXCR6^+^ CD8^+^ T cells, and IL-22–driven tumor survival. These pathways collectively create an immunosuppressive microenvironment, reducing the effectiveness of immune checkpoint inhibitors and promoting HCC progression.

Hepatocellular carcinoma, particularly in the context of NAFLD and its progressive form NASH, employs diverse strategies to evade immune surveillance. These mechanisms can be broadly categorized into three distinct yet interrelated pathways. Oncogenic activation of β-catenin (CTNNB1), frequently observed in NAFLD-related HCC, drives immune exclusion by disrupting immune cell recruitment. Specifically, CTNNB1 mutations upregulate TNFRSF19, a receptor that suppresses senescence-associated cytokines such as IL-6 and CXCL8, thereby impairing pro-inflammatory signaling required for effective immune infiltration. This immune-silent tumor phenotype can be partially reversed by Wnt inhibitors, underscoring the β-catenin/TNFRSF19 axis as a pivotal mediator of immune evasion and a promising therapeutic target (177) (**Figure 3**). Distinct metabolic features of NASH contribute to T cell dysfunction. Lipid accumulation and altered fatty acid metabolism result in apoptosis of cytotoxic CD4^+^ T cells and expansion of auto-aggressive CXCR6^+^ CD8^+^ T cells, which paradoxically promote tumor growth. These CD8^+^PD-1^+^CXCR6^+^ subsets display transcriptional and functional exhaustion profiles, contributing to a suppressive tumor microenvironment. Notably, their persistence despite ICIs therapy has been associated with poor treatment responsiveness, suggesting their role as potential resistance factors. Moreover, their unique phenotypic features and enrichment in NASH-associated HCC highlight their utility as prognostic biomarkers and immunotherapeutic targets under active investigation. These dysfunctional CD8^+^ T cells are enriched in steatohepatitic HCC (SH-HCC), which also displays CPT2 downregulation and oncometabolite accumulation, further exacerbating immune evasion. These metabolic shifts reflect a reprogrammed tumor microenvironment that suppresses effective antitumor immunity (178). Cytokine-driven immune suppression. Certain cytokines, particularly IL-22, contribute to immune evasion by modulating the tumor microenvironment. Produced by T cells and innate lymphoid cells, IL-22 has dual roles—while protective in early liver injury, it also promotes tumor cell proliferation, survival, and immune suppression in advanced disease states. Elevated IL-22 signaling in HCC has been linked to poor prognosis and reduced efficacy of immunotherapies (179). Moreover, NAFLD-HCC exhibits an altered immune landscape with lower responsiveness to ICIs, partly due to these cytokine-mediated suppressive mechanisms (180, 181) (**Table 1**).

**Table 1:** Integrated immunometabolic mechanisms driving NASH-associated hepatocellular carcinoma (HCC).

Pathophysiological axis	Molecular/cellular components	Mechanistic events	Impact on HCC development
Lipotoxicity & ROS	Free fatty acids, ROS, NLRP3 inflammasome	Lipid accumulation induces mitochondrial dysfunction and ROS; NLRP3 activation promotes IL-1β/IL-18 production	DNA damage, apoptosis–proliferation imbalance, immune activation
Macrophage Polarization	Kupffer cells, MoMFs, M1/M2 phenotypes, Galectin-12	M1 promotes inflammation (TNF-α, IL-1β), M2 enhances fibrosis via TGF-β1; Galectin-12 modulates switch	Sustained inflammation, fibrotic scaffold, tumor support
CD8^+^ & CXCR6^+^ T cells	Dysfunctional CD8^+^PD-1^+^ T cells, pro-tumor CXCR6^+^CD8^+^ cells	Chronic antigen stimulation leads to exhaustion and immune evasion	Reduced immunosurveillance, immune escape of malignant clones
Gut–Liver Axis	Microbiota, LPS, bile acids, SCFAs	Dysbiosis alters bile acid profiles, activates TLRs, disrupts FXR signaling	Promotes hepatic inflammation, barrier dysfunction, fibrosis
β-Catenin Signaling	CTNNB1 mutation, TNFRSF19	Suppresses IL-6/CXCL8, excludes T cell infiltration	Immune exclusion, ICI resistance	
HIF1A–Hypoxia Axis	HIF1A, lactate, TAMs	Induces angiogenesis, TAM M2 polarization, metabolic reprogramming	Supports tumor survival, immune evasion

### 4.3 Changes in the microenvironment

**Figure 4:**
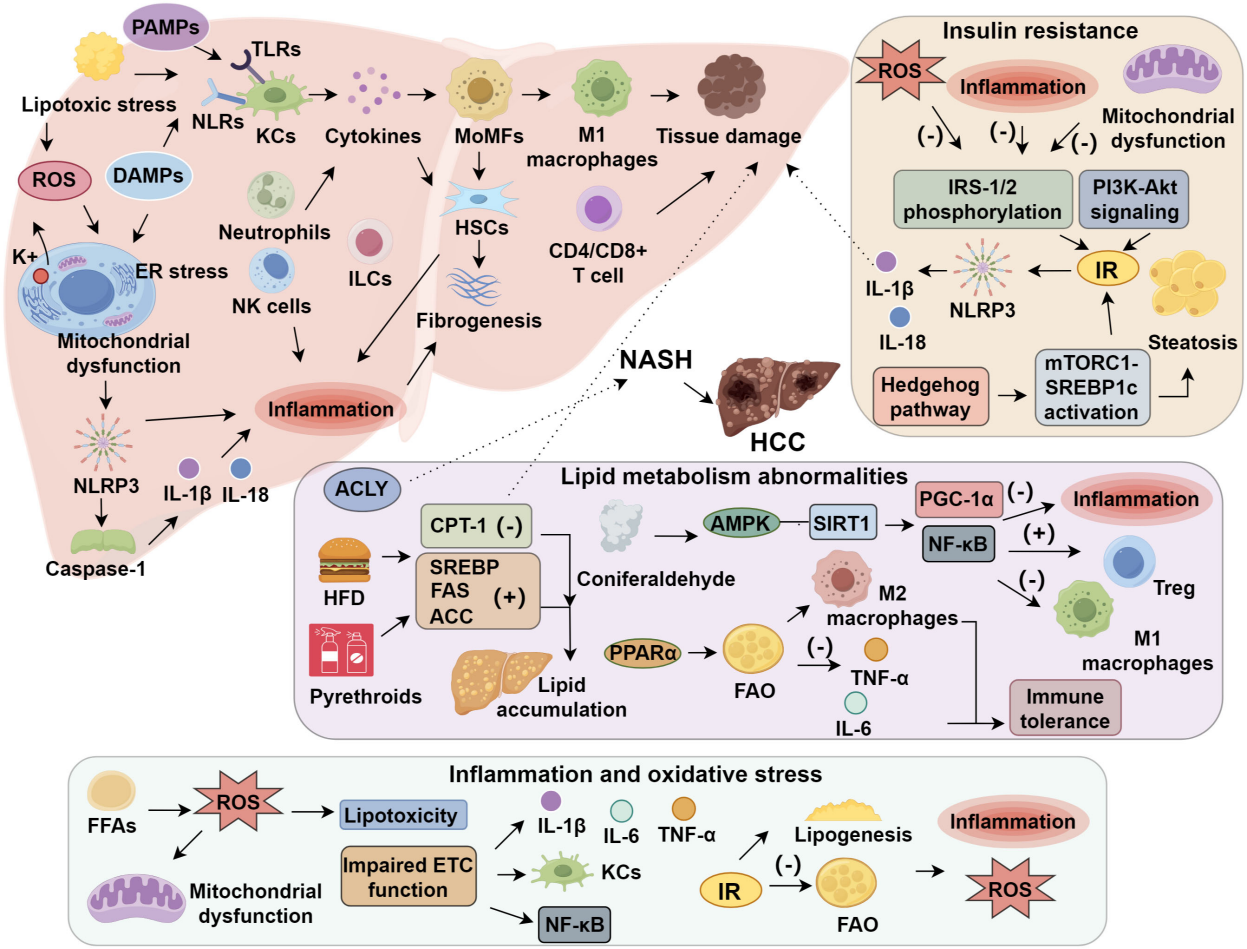
Immune cells and metabolic dysregulation in NASH-associated hepatocellular carcinoma. Chronic lipid overload induces lipotoxicity, excessive reactive oxygen species (ROS) generation, oxidative stress, and activation of the NLRP3 inflammasome, promoting hepatic inflammation and steatosis. Concurrent insulin resistance exacerbates endoplasmic reticulum (ER) stress and mitochondrial dysfunction, further amplifying inflammatory signaling. These metabolic disturbances activate resident Kupffer cells and recruit monocyte-derived macrophages, reshaping the hepatic immune microenvironment and altering T cell and natural killer (NK) cell functions. Dysregulated cytokine signaling, including increased IFN-γ, IL-1, and TGF-β, together with aberrant β-catenin signaling, drives hepatocyte malignant transformation and HCC development. In parallel, gut–liver axis disruption leads to altered bile acid composition, impaired intestinal barrier integrity, and translocation of microbiota-derived metabolites, which modulate bile acid signaling and adaptive immune responses in the liver. This integrated metabolic–immunological network culminates in immune dysfunction and tumor progression. Potential therapeutic strategies targeting these pathways include metabolic modulators (GLP-1 receptor agonists, FXR agonists, PPAR agonists, and AMPK activators), immunotherapies such as immune checkpoint inhibitors and anti-inflammatory agents, and combination therapies that concurrently modulate metabolism, gut microbiota, and antitumor immunity.

The microenvironment of NASH, a progressive type of NAFLD, significantly influences the pathophysiology and development of HCC. Chronic inflammation, hepatocyte damage, fibrosis, and metabolic dysregulation are the hallmarks of NASH, and they all work together to create an environment that is conducive to tumor growth. The prolonged inflammatory state caused by immune cell infiltration and activation is a key characteristic of the NASH microenvironment. Macrophages, especially those surrounding dead hepatocytes in crown-like formations, exhibit unique gene expression patterns that support tumor growth and fibrosis induced by metabolic stress (182). Together with activated fibroblasts that drive cancer-related pathways, these NASH-specific macrophages create a favorable environment for hepatocarcinogenesis. The presence of immunosuppressive cell subsets, such as regulatory T cells (Tregs), which impede efficient antitumor immune responses and facilitate tumor immune evasion, further complicates the immunological landscape in NASH-related HCC (183). Furthermore, it has been shown that metabolic changes in the NASH milieu, such as dysregulated lipid metabolism and cholesterol accumulation, affect the activity of natural killer T (NKT) cells, compromising antitumor immunosurveillance and promoting tumor growth (184). Hepatocyte-derived hypoxia-inducible factor 1-alpha (HIF1A) activation during NASH progression results in metabolic reprogramming, angiogenesis, and immunosuppressive macrophage polarization, all of which promote tumorigenesis (185). Hypoxia itself emerges as a pivotal determinant of the NASH tumor microenvironment. Moreover, interactions between microenvironmental factors and genetic alterations—such as CTNNB1 mutations frequently observed in NAFLD-HCC—contribute to immune evasion by suppressing senescence-associated secretory phenotypes and promoting immune exclusion (177). Perturbations in gut microbiota composition and metabolite production also modulate hepatic immunity, fibrosis, and inflammation, thereby expediting the transition from metabolic-dysfunction-associated steatotic liver disease to HCC (186, 187). Extracellular matrix (ECM) remodeling, a hallmark of NASH fibrosis, further reinforces tumor progression by offering structural support for invasion and metastasis (188). Notably, the immunosuppressive microenvironment may underlie the suboptimal efficacy of immune checkpoint blockade in NAFLD-HCC; however, combinatorial regimens incorporating anti-angiogenic or metabolic modulators may enhance clinical outcomes (189, 190) (**Figure 4**).

## 5 Advances in therapeutic strategies

### 5.1 Emerging roles of immunotherapy in NASH-associated HCC

The accumulation of dysfunctional CD8^+^PD-1^+^ T cells in NASH paradoxically exacerbates liver injury upon PD-1 blockade, complicating immunotherapeutic outcomes in this context (191). Nevertheless, recent clinical data reveal that patients with NAFLD-associated HCC derive enhanced objective response rates and progression-free survival from combination regimens pairing immune checkpoint inhibitors (ICIs) with anti-angiogenic agents, notably the atezolizumab–bevacizumab protocol (189, 192). Furthermore, it has been suggested that altering metabolic pathways and the gut microbiome might improve the effectiveness of immunotherapy. The gut–liver axis plays a pivotal role in sculpting hepatic immune dynamics, and dysbiosis characteristic of NAFLD/NASH has been shown to modulate tumor progression and immune responses. Therapeutic modulation of microbial composition or host metabolism may recondition the tumor microenvironment, thereby enhancing ICI responsiveness (193, 194). Recent studies also emphasize the contribution of new immune cell subsets to the immunological landscape in chronic liver disease and HCC, including tissue-resident memory T cells and γδT cells. To minimize liver damage and reestablish efficient antitumor immunity, these cells might be targeted for precision immunotherapy (195, 196). The immune-related transcriptional signatures—such as estrogen pathway–associated gene panels—have been developed to stratify patients by predicted immunotherapy responsiveness, thus enabling more personalized treatment strategies (197). Despite these advancements, optimizing immunotherapy for NASH-related HCC remains challenging. Heterogeneity in immune dysfunction, metabolic rewiring, and susceptibility to immune-mediated hepatic damage necessitates careful patient selection and tailored combinatorial approaches. Ongoing clinical trials are actively evaluating ICI efficacy in diverse HCC subpopulations, including those with underlying metabolic liver disease, and are testing regimens incorporating anti-angiogenic agents, metabolic modulators, and microbiome-based interventions (198–200). Furthermore, the metabolic reprogramming and chronic inflammation characteristic of NASH-HCC contribute to T cell exhaustion and impaired persistence of adoptively transferred cells (201, 202). The fibrotic stroma, elevated levels of inhibitory cytokines (IL-10, TGF-β), and lipid accumulation within the TME further hinder the expansion, trafficking, and cytotoxic function of engineered T cells (203, 204). These microenvironmental constraints underscore the need to optimize CAR-T/TCR strategies for liver cancer by incorporating metabolic modulators, improving antigen specificity, and targeting immunosuppressive cues unique to NASH-related HCC.

### 5.2 Metabolic regulatory drugs

With the development of new metabolic regulatory medications targeting key pathways in lipid metabolism, inflammation, and fibrosis, the therapy landscape for NASH, a progressive variant of NAFLD, is undergoing significant transformation. These medications seek to address the underlying metabolic abnormalities that cause hepatic damage and steatosis. Among them, agonists of the peroxisome proliferator-activated receptor alpha (PPARα) have attracted significant interest due to their critical role in regulating lipid oxidation and reducing hepatic inflammation. To promote lipid oxidation and mitigate steatosis, recent research has proposed new selective PPARα modulators, such as the DY series, which have shown effectiveness in upregulating PPARα target genes implicated in NASH pathogenesis (205). In addition, it has been demonstrated that a crucial mechanism for promoting fatty acid β-oxidation is the SIRT1/PGC-1α/PPARα signaling axis. In NASH models, a natural substance called formononetin improves hepatic steatosis and lipid metabolism by activating this system (206). Activation of the farnesoid X receptor (FXR)—a bile acid–sensing nuclear receptor that governs bile acid, lipid, and glucose metabolism—exerts suppressive effects on hepatic steatosis and fibrosis. Obeticholic acid (OCA), the first FXR agonist to reach clinical trials for NASH, demonstrated modest hepatoprotective efficacy; however, its clinical utility has been constrained by adverse events such as pruritus and dyslipidemia (207). Despite these challenges, ongoing efforts are focused on refining FXR-targeted therapies through the development of more selective agonists and combinatorial strategies to enhance efficacy while mitigating toxicity (207, 208). Notably, the gut microbiota–FXR axis has emerged as a compelling therapeutic avenue, as microbial modulation of bile acid composition profoundly influences FXR signaling, thereby shaping systemic metabolism and the trajectory of NAFLD progression (209).

The endocrine roles of fibroblast growth factors (FGFs), particularly FGF19 and FGF21, in regulating lipid and glucose metabolism have recently garnered attention. Clinical trials of FGF-based therapies have demonstrated promising efficacy in alleviating steatosis, inflammation, and fibrosis in patients with NAFLD (210). Enhancing insulin sensitivity and increasing energy expenditure are two of these medicines’ pleiotropic effects, which are essential for metabolic correction in NASH. Hepatocyte-expressed G protein-coupled receptors (GPCRs) are also desirable targets for drugs because of their regulatory functions in liver metabolism. The liver is thought to harbor more than 50 GPCRs, and altering these receptors might affect lipid and glucose balance, opening new treatment options for NASH (211). Additionally, targeting cholesterol transporters such as NPC1L1, implicated in cholesterol absorption and NASH progression, has been proposed to rebalance cholesterol metabolism (212). Activation of AMPK, a master regulator of cellular energy status, offers dual benefits by promoting lipid catabolism and reducing oxidative stress. Gossypetin, a naturally derived flavonoid, attenuates hepatic steatosis, inflammation, and fibrosis in NASH models through both antioxidant activity and AMPK activation (213). Similarly, GLP-1RAs ameliorate hepatic lipid accumulation by modulating lipid turnover and inducing autophagy via the AMPK/SIRT1 axis (214). Preclinical research suggests that peripheral serotonin receptor antagonists targeting 5-HT2A receptors may decrease hepatic fat accumulation without adverse effects on the central nervous system (215). Natural compounds such as brown algae–derived plant polysaccharides have shown hepatoprotective properties, including modulation of gut microbiota, suppression of hepatic inflammation, and regulation of lipid metabolism, offering low-toxicity adjunctive options for NASH intervention (216–218).

Recent findings indicate that exosomes derived from mesenchymal stem cells (MSCs) can ameliorate hepatic steatosis by enhancing fatty acid oxidation and suppressing lipogenesis through CAMKK1-mediated activation of AMPK, offering a novel biologic strategy for NASH treatment (219). Despite such promising advances, the development of safe and effective metabolic therapies for NASH remains challenged by the disease’s complex pathophysiology, characterized by intertwined fibrotic, inflammatory, and metabolic signaling networks. To address this complexity, combinatorial therapeutic strategies—targeting multiple pathogenic axes either concurrently or in sequence—are increasingly being explored to maximize efficacy while minimizing adverse effects (220). Furthermore, leveraging precision medicine approaches, including patient-specific genomic, epigenomic, and microbiome profiling, holds substantial promise for tailoring metabolic therapies to individual disease phenotypes, thereby enhancing therapeutic responsiveness and long-term outcomes (221, 222).

### 5.3 Nutritional interventions and lifestyle modifications

In the continued absence of approved pharmacological therapies, lifestyle interventions remain the primary strategy for managing NAFLD and its progressive form, NASH. Robust evidence indicates that weight loss achieved through dietary modification and enhanced physical activity ameliorates hepatic steatosis, inflammation, and even fibrosis, thereby reducing the progression to cirrhosis and HCC (223, 224). Given the frequent coexistence of cardiometabolic disorders in NAFLD, dietary interventions such as the Mediterranean diet—rich in monounsaturated fats, fiber, and antioxidants—have demonstrated beneficial metabolic effects independent of caloric restriction, improving both liver fat accumulation and cardiovascular risk profiles (225, 226). Alternative dietary strategies, including intermittent fasting, the Dietary Approaches to Stop Hypertension (DASH) diet, and ketogenic regimens, have also yielded encouraging results in improving hepatic histology and metabolic homeostasis (225). Physical activity, encompassing both aerobic and resistance training, enhances insulin sensitivity and contributes to weight reduction, both critical factors in NAFLD pathogenesis. Importantly, these lifestyle modifications not only reduce hepatic lipid burden but also mitigate the risk of type 2 diabetes and cardiovascular disease—the principal causes of mortality in patients with NAFLD (227, 228).

Genetic variables, such as the PNPLA3 rs738409 polymorphism, which alters responsiveness to diet and exercise regimens, might affect the success of lifestyle interventions, underscoring the need for individualized methods (229). In pediatric populations, early lifestyle interventions focusing on low-fat, low-sugar diets and increased physical activity are critical for halting NASH progression and improving both metabolic and psychosocial outcomes (230, 231). However, long-term adherence to lifestyle modifications remains challenging, with fewer than half of patients achieving sustained weight loss sufficient to improve hepatic histology (232). With promising results in weight reduction and metabolic improvements, emerging endoscopic bariatric procedures offer less-invasive alternatives to surgery and may help alleviate NAFLD/NASH (232, 233). Specific bioactive dietary components, including branched fatty acid esters of hydroxyl fatty acids (9-PAHSA), have shown hepatoprotective and anti-inflammatory effects in animal models, suggesting possible adjuvant functions for nutritional supplements beyond weight reduction (223). The complex benefits of diet and exercise are further supported by the fact that lifestyle changes affect intestinal barrier integrity and gut microbiota composition, which are increasingly recognized as essential modulators of NAFLD development and hepatocarcinogenesis (187). Lifestyle interventions contribute to improved health-related quality of life, particularly in domains related to physical functioning (226, 234, 235).

## 6 Future research directions

### 6.1 New immune targets

Given the intricate interactions between immune dysregulation and metabolic abnormalities that propel disease progression and the development of HCC, the investigation of novel immune targets in the setting of NASH is essential for developing treatment approaches. The liver’s distinct immune milieu, which includes a wide range of innate and adaptive immune cells such as T cells, natural killer (NK) cells, macrophages, dendritic cells, and liver-resident cells, including Kupffer cells and hepatic stellate cells, has been highlighted in recent research. In NASH and associated liver malignancies, these cells support the immunosuppressive microenvironment that promotes carcinogenesis, fibrosis, and chronic inflammation (236). Targeting immunological checkpoints and signaling pathways that regulate immune cell activation and fatigue is one potential approach. For example, cytotoxic T-lymphocyte-associated protein 4 (CTLA-4) and the PD-1/PD-L1 axis have been linked to the compromised anti-tumor immune responses seen in NASH-associated HCC, even though clinical responses to ICIs in this context are still inconsistent and occasionally suboptimal (190, 237). This variation underscores the need to identify new immune modulators and combinatorial approaches tailored to the inflammatory and metabolic environment of NASH. MDSCs, which proliferate in chronic liver disease and have potent immunosuppressive effects, are implicated in the development of immunotherapy resistance and disease progression, according to new research. An innovative immunotherapeutic approach to effectively restore anti-tumor immunity in NASH-HCC is to target MDSCs and the signaling pathways that are linked to them (238).

Recent studies have identified novel immune receptors, such as signaling lymphocytic activation molecule family 1 (SLAMF1), as key mediators of hepatocyte death, positioning them as potential biomarkers for NASH and therapeutic targets to mitigate immune-driven hepatic injury (239). Targeting immunometabolism may improve therapeutic efficacy because immune cell function and phenotype are also influenced by metabolic reprogramming in the NASH liver microenvironment, characterized by altered glycolysis, oxidative phosphorylation, and lipid metabolism (240).

Extracellular vesicles (EVs) and toll-like receptor 4 (TLR4) signaling are important in regulating inflammation and immune responses in NASH and HCC (241). The gut–liver axis, along with microbiota-derived metabolites, has been shown to modulate intrahepatic immune responses, suggesting that microbiota-targeted therapies may indirectly reprogram immune dynamics in NASH (242). Kinase inhibitors may act as supplemental immune modulators, and protein kinases implicated in metabolic and inflammatory signaling pathways are being recognized as critical molecular contributions to the development of HCC and NASH pathophysiology (243). Preclinical models of NASH-HCC have demonstrated that agents such as 6-gingerol can potentiate anti-tumor immunity by reprogramming tumor-associated macrophages toward a pro-inflammatory M1 phenotype via the NOX2/Src/MAPK axis (244). Moreover, epigenetic regulation has emerged as a novel target; for instance, METTL3 influences immune infiltration and activation by modulating m6A RNA methylation and cholesterol biosynthesis, thereby providing a route to counter immune evasion in NASH-related HCC (245).

### 6.2 Metabolic and immune crosstalk research

A comprehensive understanding of the pathophysiology and progression of NAFLD and its advanced form, NASH, necessitates elucidation of the intricate crosstalk between metabolic and immune networks (246, 247). This bidirectional interplay governs immune cell activation, disrupts metabolic equilibrium, and drives hepatic microenvironmental remodeling—hallmarks underpinning disease advancement and the eventual emergence of HCC (132, 137, 248). Recent insights from multi-omics and single-cell transcriptomic analyses have delineated how host metabolic circuitry intersects with immunological pathways (249, 250). In particular, gut microbiota-derived metabolites have emerged as pivotal regulators of intrahepatic immune tone and metabolic homeostasis (251, 252). Perturbations in host–microbiota dynamics—culminating in impaired mitophagy—engender a proinflammatory hepatic niche that accelerates NAFLD progression (253). Notably, transcriptional alterations in genes governing energy metabolism and immune modulation—across macrophages, monocytes, hepatocytes, and endothelial cells—are linked to the microbial shift from symbiotic taxa (Escherichia coli in healthy individuals) to pathogenic lineages (Sphingomonadales) in NAFLD and NASH (253). The disruption of host–bacteria interactions involving key mitophagy regulators such as SQSTM1, OPTN, and BNIP3L in NASH exemplifies how metabolic collapse and immune dysfunction synergistically amplify hepatic inflammation (253, 254).

Gut microbiota-derived metabolites—including short-chain fatty acids (SCFAs), secondary bile acids (BAs), and tryptophan derivatives—serve as key signaling mediators that modulate immune cell function and hepatic metabolic circuits. SCFAs, for example, promote immunological tolerance and energy homeostasis through G-protein-coupled receptors, whereas dysbiosis-associated alterations in BA profiles perturb farnesoid X receptor (FXR) signaling, disrupting metabolic and immune equilibrium. Perturbation of BA metabolism and downstream signaling cascades contributes to hepatic steatosis, inflammation, and fibrosis, underscoring the dual immunometabolic regulatory roles of these pathways in liver disease (255–257). In NAFLD, immune cells, such as macrophages, T cells, and B cells, undergo cellular metabolic reprogramming that affects their activation, polarization, and effector functions. The altered lipid metabolism and programmed cell death pathways observed in macrophages in the liver and adipose tissue influence inflammatory responses and lead to persistent low-grade inflammation, a hallmark of metabolic liver disease. Metabolic changes determine the pro- or anti-inflammatory phenotypes of T cells, and CD4^+^ T cell development and function are significantly influenced by lipid metabolism. B cells, through antibody production and cytokine secretion, also participate in orchestrating hepatic immunometabolism and energy regulation (258–260).

Nucleotide-binding domain leucine-rich repeat receptors (NLRs) and the cGAS-STING pathway are examples of intracellular innate immune sensors that combine immune activation with metabolic signals. Mitochondrial stress and metabolic imbalance stimulate the cGAS-STING axis, which sets off proinflammatory cascades that worsen insulin resistance and the advancement of non-alcoholic fatty liver disease. NLR inflammasomes affect immunological responses and metabolic balance through interactions with the gut microbiota and metabolic pathways (261–263). Moreover, nuclear receptors, such as peroxisome proliferator-activated receptors (PPARs) and FXR, operate as molecular links between immune control, metabolism, and gut microbiota. These receptors coordinate the gut-liver axis and maintain hepatic homeostasis by regulating gene expression associated with immune cell activation, autophagy, and lipid and glucose metabolism. Their therapeutic potential is highlighted by the fact that dysregulation of these nuclear receptor pathways leads to immune-mediated liver damage and metabolic dysfunction (256, 264).

Therapeutic approaches that target the metabolic-immune interface are being investigated. These include pharmaceutical drugs that target immunological checkpoints and metabolic pathways, as well as modifications to gut microbiota composition via probiotics, prebiotics, and fecal microbiota transplantation (FMT). Compounds like digoxin and helveticoside, which influence metabolic reprogramming and immune cell infiltration, have demonstrated therapeutic potential in NAFLD models. Novel targets such as TM4SF5, a regulator of immune cell interactions and nutrient transport in hepatocytes, further expand the therapeutic landscape. Hepatic inflammation and fibrosis may be reduced by modifying the NLRP3 inflammasome and cGAS-STING signaling (85, 265, 266).

In sum, the pathogenesis of NAFLD and related liver diseases arises from tightly interwoven metabolic and immune dysregulation, orchestrated through host–microbiota interactions and cellular reprogramming. Elucidating these interconnected pathways paves the way for integrated therapeutic strategies that target both arms of the disease process, offering a rational framework to curb disease progression and improve clinical outcomes (251, 267) (**Table 2**).

**Table 2:** Therapeutic strategies for NASH-associated hepatocellular carcinoma (HCC).

Therapeutic strategy	Agents	Primary targets	Mechanism	Preclinical/clinical status	Challenge
Immunotherapy (ICI)	Anti–PD-1 (nivolumab), Anti–CTLA-4, Atezolizumab + Bevacizumab	PD-1/PD-L1, CTLA-4, VEGF	Restores T cell cytotoxicity, inhibits angiogenesis	Approved for HCC; lower efficacy in NASH-HCC	CD8^+^ T cell exhaustion, paradoxical liver injury
Macrophage Reprogramming	6-Gingerol, CSF1R inhibitors	NOX2/Src/MAPK, CSF1R	Promotes M1 polarization, reduces tumor-supportive M2 TAMs	Preclinical	Specificity, delivery, systemic effects
Metabolic Modulators	GLP-1RAs (semaglutide), PPARα/γ agonists, FXR agonists (obeticholic acid), AMPK activators (metformin, gossypetin)	Lipogenesis, FAO, glucose metabolism	Reduces steatosis, improves insulin resistance, inhibits fibrosis	Phase II/III trials for NASH, some off-label in HCC	Safety (pruritus with OCA), resistance, liver function constraints
Microbiota-Targeted	Probiotics, FMT, antibiotics, BA analogues	Gut-liver signaling, FXR, bile acid homeostasis	Restores microbial diversity, modifies bile acid–immune crosstalk	Exploratory/early trials	Individual variability, durability
Epigenetic Modulators	METTL3 inhibitors	m6A methylation, cholesterol biosynthesis	Reduces tumor cell proliferation and immune suppression	Preclinical	Off-target effects, delivery systems
Combination Strategies	ICI + anti-VEGF; ICI + FXR agonist; ICI + metabolic modulator	Dual immune–metabolic targets	Synergistic antitumor + anti-inflammatory effects	Ongoing trials (IMbrave150)	Timing, toxicity, patient selection

## 7 Conclusion

Hepatic inflammation, fibrogenesis, and carcinogenesis are all fueled by the intricate, reciprocal interactions between immune dysregulation and metabolic dysfunction, as evidenced by the expanding body of studies on NASH-associated HCC. In the past, immunological disruptions involving T cells, macrophages, and natural killer cells, as well as metabolic changes such as insulin resistance, lipid buildup, and mitochondrial dysfunction, were studied separately. Emerging data, however, show that they are interdependent: immune cells worsen metabolic stress and fibrosis, while metabolic disturbances alter immune cell morphologies, resulting in a vicious cycle of liver damage and tumor development. This convergence not only reveals new therapeutic vulnerabilities but also advances our mechanistic understanding of disease development. Despite these developments, finding biomarkers and generalizing treatments is made more difficult by the significant variability in patient immune-metabolic profiles. The inability of preclinical models to accurately replicate the complexity of human disease underscores the need for integrated systems biology approaches, such as multi-omics and longitudinal research, to elucidate the spatiotemporal dynamics of the hepatic microenvironment.

To accelerate clinical translation, future studies should focus on identifying metabolic signatures and circulating biomarkers that can predict response to immunotherapies, particularly immune checkpoint inhibitors. Furthermore, targeting specific immunometabolic axes—such as PPARα-mediated macrophage polarization, lactate-driven M2-like TAM programming, or SIRT1/PGC-1α signaling—holds promise for restoring immune surveillance and rebalancing hepatic metabolism. Equally important is the integration of microbiome-based strategies, including fecal microbiota transplantation and metabolite modulation (SCFAs, bile acids), to reshape the gut–liver–immune axis and enhance therapy responsiveness. To restore homeostasis and anti-tumor immunity, combinatorial strategies—which mix metabolic modulators with immunotherapies like checkpoint inhibitors have gained attention due to the therapeutic awareness of immune-metabolic interaction. It’s still difficult to optimize these regimens, especially when hepatic impairment is present in advanced illness. Ultimately, a precision medicine framework that integrates immune, metabolic, and microbiome-derived data will be essential for stratifying patients and tailoring therapeutic interventions. To improve outcomes in NASH-associated HCC and advance precision medicine, a multidisciplinary, patient-specific paradigm that incorporates immunological and metabolic insights will be crucial.
